# Emergence of Moiré Dirac Fermions at the Interface of Topological and 2D Magnetic Insulators

**DOI:** 10.1021/acsnano.5c10193

**Published:** 2025-10-10

**Authors:** Ilya I. Klimovskikh, Sebastien E. Hadjadj, Amitayush Thakur, Aymeric Saunot, Celia Rogero, Massimo Tallarida, Ji Dai, Vesna Mikšić Trontl, Andrew P. Weber, Genda D. Gu, Jorge Lobo-Checa, Maxim Ilyn, Tonica Valla

**Affiliations:** † 226245 Donostia International Physics Center (DIPC), Donostia-San Sebastián 20018, Spain; ‡ 9166 Centro de Física de Materiales CSIC-UPV/EHU, Donostia-San Sebastián 20018, Spain; § Departamento de Polímeros y Materiales Avanzados, Universidad del País Vasco UPV/EHU, Donostia-San Sebastián 20018, Spain; ∥ Université Paris-Saclay, CNRS, Institut des Sciences Moléculaires d’Orsay, 91405 Orsay, France; ⊥ 16379 ALBA Synchrotron Light Source, Cerdanyola del Vallès, 08290 Barcelona, Spain; # Institut za Fiziku, Bijenička 46, HR-10000 Zagreb, Croatia; ∇ ICFO-Institut de Ciencies Fotoniques, The Barcelona Institute of Science and Technology, Castelldefels, Barcelona 08860, Spain; ○ Condensed Matter Physics and Materials Science Department, 153963 Brookhaven National Laboratory, Upton, New York 11973, United States; ◆ Instituto de Nanociencia y Materiales de Aragón (INMA), CSIC-Universidad de Zaragoza, Zaragoza 50009, Spain; ¶ Departamento de Física de la Materia Condensada, Universidad de Zaragoza, Zaragoza E-50009, Spain

**Keywords:** moiré materials, topological insulators, 2D magnets, ARPES, STM, Dirac cone, transition metal dihalides

## Abstract

Dirac Fermions on the surface of the topological insulator are spin-momentum locked and topologically protected, making them interesting for spintronics and quantum computing applications. When in proximity to magnetism and superconductivity, these electronic states could result in quantum anomalous Hall effect and Majorana Fermions, respectively. An even more dramatic enrichment of the topological insulators’ physics is expected for moiré superlattices, where, analogously to the twisted graphene layers, electronic correlations could be strongly enhanced, a task previously notoriously difficult to achieve in topological matter. Until now, the experimental confirmation of such moiré properties has remained elusive. Here, we grow the two-dimensional van der Waals magnetic insulators FeX_2_ (where X = Cl or Br) on top of the topological insulator Bi_2_Se_3_ and establish a moiré superlattice formation at the interface. By means of scanning tunneling microscopy and angle-resolved photoemission spectroscopy, we investigate the electronic properties of the formed moiré superlattice and demonstrate its tunability via the film choice. We reveal replicated Dirac cones and focus on their intersections, which, in the case of FeBr_2_/Bi_2_Se_3_, occur below the Fermi level. We identify the signatures of small gaps at the intersections around the M̅_*i*_ points that we attribute to the moiré interaction. These findings point to the specific type of magnetic moiré potential that breaks the time-reversal symmetry at these points but not at the Γ̅ point. Our observations provide an intriguing scenario of correlated topological phases induced by moiré superlattice that may result in topological superconductivity, high Chern number phases, and exotic noncollinear magnetic textures.

## Graphical abstract

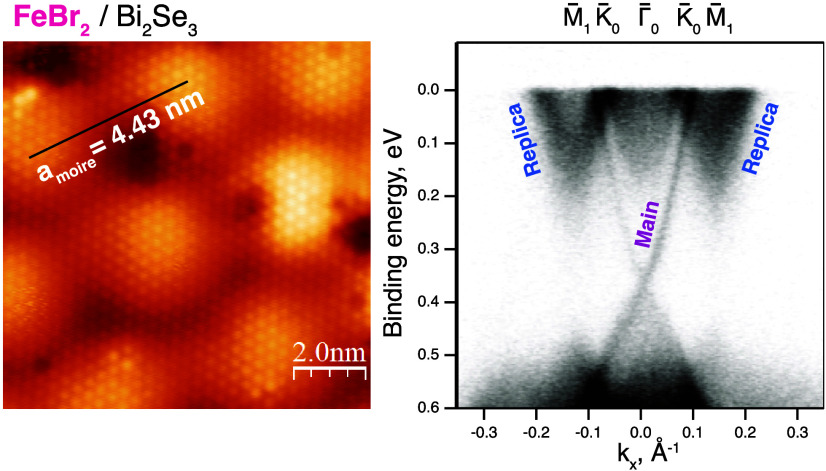

When two layers of van der Waals (vdW) materials are stacked with a twist angle and/or a lattice mismatch, a moiré superstructure arises. An explosive research interest in twistronics has been ignited by the fabrication and characterization of the magic-angle graphene bilayers.^1,2^ Moiré superpotential replicates the Dirac cone of graphene and opens minigaps, leading to flat bands around a twist angle of ≈1°. This gives rise to an incredibly rich physics of strongly correlated electrons in two dimensions, resulting in unconventional superconductivity, Mott insulating phases, and quantum anomalous Hall effect.^1–6^ Besides graphene, semiconducting moiré heterostructures realized in transition metal dichalcogenides are of great interest, where Wigner crystal states and Hubbard model physics have been reported.^7–11^ The recent studies on moiré two-dimensional (2D) vdW magnets have been limited to twisted CrI_3_ ^12–14^ layers, with the observation of noncollinear magnetic structures.

Theoretically, even more intriguing physics is expected at the moiré modulated surfaces of topological insulators (TIs). The Dirac dispersion should get renormalized, potentially resulting in topological superconductivity.^15,16^ However, contrary to graphene, spin-momentum locked Dirac Fermions remain degenerate at the crossings occurring at the protected M̅ points of the mini-zone boundaries, with gaps opening at other, unprotected crossings, forming van Hove singularities.^17,18^ Nevertheless, the time-reversal symmetry can be broken either by strong coulomb interactions, leading to Meron-like textures at the surface,^19^ or by inducing magnetism at the interface, yielding high-order Chern insulator and other exotic phases.^15,16,19–25^ To the best of our knowledge, an experimental demonstration of this effect is still lacking. The moiré superlattices on surfaces of TIs have not been realized in a controllable way, and their electronic properties are yet to be explored.^26–33^

Here, we achieve and study moiré heterostructures consisting of a single slab of the 2D vdW magnet FeX_2_ (X = Cl or Br) directly grown on the surface of the topological insulator Bi_2_Se_3_ (BS). Single-unit-cell-thick films of transition metal dihalides (TMDHs) have recently been shown to be 2D vdW ferromagnets with a relatively large band gap (3–5 eV).^34–36^ Due to the lattice mismatch, we find that the growth of TMDHs on BS results in moiré patterns with a tunable periodicity (2.9–4.4 nm), determined by the choice of TMDH film. The scanning tunneling microscopy (STM) measurements clearly demonstrate superlattice formation with topographic symmetry modulation depending on the applied bias voltage. Notably, angle-resolved photoemission spectroscopy (ARPES) uncovers replicated Dirac cones in the band structure. Analysis of the spectra reveals broadening of the peak at the Umklapp crossings at the M̅_*i*_ points that could be a result of the breaking of the time-reversal symmetry induced by the magnetic moiré superlattice.

In contrast to twisted graphene and other trivial 2D materials, such intrinsic magnetic field could turn out to be a valuable tool for tuning correlations in topological insulators with 2D vdW magnetic insulators, thus opening promising avenues for tunable physics shaped by the moiré superlattices.

## Results and Discussion

The growth mode of TMDHs on BS substrate is close to Frank van der–Merwe-type (details in the Experimental Methods section), similar to the growth on Au(111)^34^ or Bi(111)^35^ substrates. In Figure 1a, an STM overview image of half-monolayer coverage of FeCl_2_ (FC) on BS is shown. It can be seen that on BS large, monolayer thick islands of FC are grown with the second layer occasionally observed near the centers of the monolayer islands, far from their edges. On the FC island, a pronounced periodic modulation with a period of 2.88 nm is clearly resolved. This modulation originates from the formation of the moiré pattern and corresponds to an 8 × 8 (7 × 7) superstructure relative to FC (BS) lattice, agreeing nicely with the in-plane lattice parameters of 3.64 Å for FC and 4.15 Å for BS. The same superstructure is determined in low-energy electron diffraction patterns (see Supporting Information (S.I.)).

**Figure 1:**
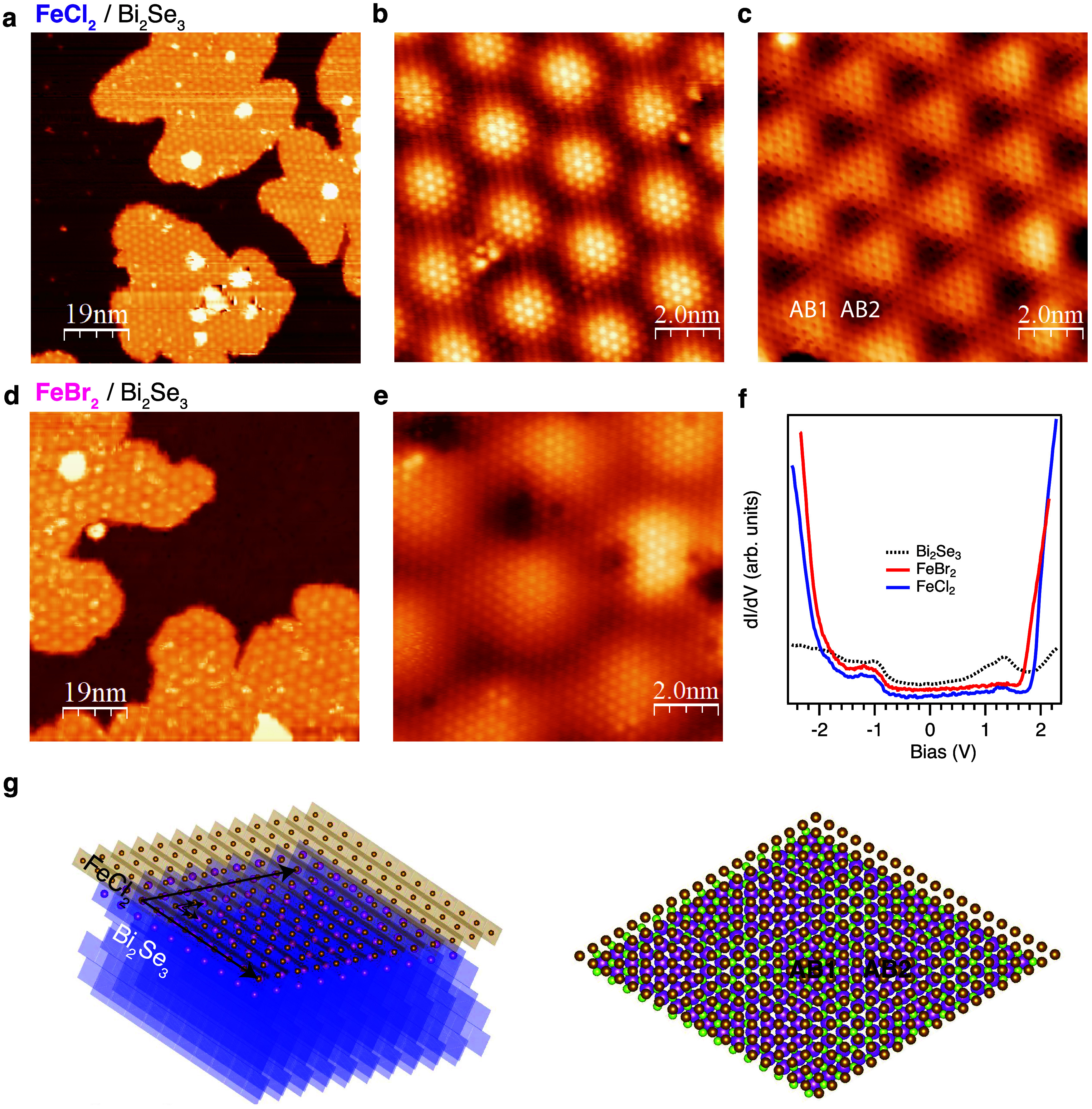
High-resolution STM/STS of FC and FB films on BS. (a) Topographic STM overview of the 0.5–0.6 ML sample of FC/BS. (b, c) Atomic-resolution STM images of the FC monolayer islands on BS at two selected bias voltages (−1.0 and −0.25 V). (d) Same as (a) for FB/BS. (e) Same as (b) for FB/BS. The extracted lattice constant of the outermost Cl (Br) layer is 3.64 ± 0.06 Å (3.80 ± 0.05 Å), and the moiré pattern has a periodicity of 2.88 ± 0.02 nm (4.43 ± 0.12 nm). (f) Average STS measurements of BS surface and FeX_2_ islands after Dirac point energy alignment (the FB STS required a rigid shift of +0.15 V, as explained in the S.I.). Note that the spectra were vertically offset for improved visualization. The STM measurements were performed at 4.9 K. STM parameters: (a, d) *U*_Bias_ = 1.6 V, *I*_Tunneling_ = 5 pA; (b) *U*_Bias_ = −1 V, *I*_Tunneling_ = 50 pA; (c) *U*_Bias_ = −0.25 V, *I*_Tunneling_ = 50 pA; (e) *U*_Bias_ = −0.1 V, *I*_Tunneling_ = 60 pA. (g) Schematic representation of the heterostructure. On the left, only the topmost Bi atoms (magenta) and Fe atoms (brown) are shown by spheres. On the right, Fe atoms, topmost Bi and Se atoms (green) are represented in order to demonstrate the different registries.

Atomically resolved STM images taken at various bias voltages are shown in Figure 1b,c. In Figure 1b, we observe the hexagonal lattice of chlorine atoms on the surface, modulated by an 8 × 8 superpotential that also has hexagonal symmetry. However, reducing the bias voltage toward the Dirac cone of BS results in changing the symmetry to trigonal, as seen in Figure 1c. This observation highly contrasts the behavior observed in twisted graphene bilayers and can be explained by different registries of AB1 and AB2 regions (see Figure 1g) due to the trigonal symmetries of the BS and FC lattices. Similar trigonal modulation was observed in moirés of transition metal dichalcogenides,^7,10^ where this morphological corrugation has been suggested to be responsible for the rich Hubbard model physics in these superlattices.^8^ Changing the film composition to FeBr_2_ (FB) results in the enlargement of the moiré periodicity to 4.43 nm, as determined from Figure 1d, in accordance with the decrease of the lattice mismatch between the substrate and film (3.8 Å). In this case, we do not observe the trigonal modulation of the moiré STM signal (see Figure 1e), which could be related to the different interaction of the film with the substrate.

To gain a deeper insight into the electronic structure of the fabricated heterostructure, differential conductance spectra (scanning tunneling spectroscopy (STS) measurements) were performed on the samples (Figures 1f and S2 and S3 in the S.I.). The spectrum of the BS substrate (black curve) is well known and represents the pronounced bulk bands and topological surface states within the bulk band gap.^29^ The spectrum taken on the FC island (blue curve) shows an attenuation of the substrate features and the formation of dominant peaks below −2 V and above 2 V, corresponding to the valence and conduction bands of the FC monolayer, revealing the large band gap of a monolayer of TMDH. STS spectrum taken on the monolayer FB island (red curve) demonstrates similar features but with a slightly smaller band gap. In these conductance spectra acquired at the TMDHs, weak in-gap features are visible, which are attributed to attenuated contributions from the substrate bulk states. We have distinguished similar TMDH- and BS-related states in the electronic structure using the resonant photoemission spectroscopy, as shown in the Supporting Information in Figure S4.

The exotic electronic properties of conventional 2D moiré materials originate from the shrinking of the Brillouin zone, replication of the bands, and emergence of avoided crossings.^37,38^ To look for these features, we study the band structure of our FeX_2_/BS interfaces by means of ARPES, which is shown in Figure 2. We present the data for two BS samples covered by a different TMDH monolayer, FC/BS (a) and FB/BS (b) (Note that the real coverages are slightly lower than one ML, see discussion below). Because of the large band gap of the TMDHs, the signal from the topological insulator is virtually unaltered, aside from being attenuated by the film, and it always dominates the low-energy region of the ARPES spectra. Both band structures (a and b) clearly show the pronounced Dirac cone at the Γ̅_0_ point and additional Dirac cones (replicas) laterally shifted by a wave vector of 0.25 Å^–1^ (FC) and 0.17 Å^–1^ (FB). These values match the inverse lattice vectors *b*_*M*_ = 4π/(√3*a*_*M*_) of the moiré superstructures (*a*_*M*_ = 2.9 nm (FC) and *a*_*M*_ = 4.4 nm (FB), respectively) determined by STM in Figure 1, confirming that ARPES and STM data are experimentally correlated. In addition to topological surface bands, a set of quantized states (QWSs) of valence and conduction bands emerge around the Γ̅_0_ point, for both systems. These quantized states usually appear due to the band bending or by doping and aging of the BS surface, and are related to the uncovered parts of the surface.^39–41^

**Figure 2:**
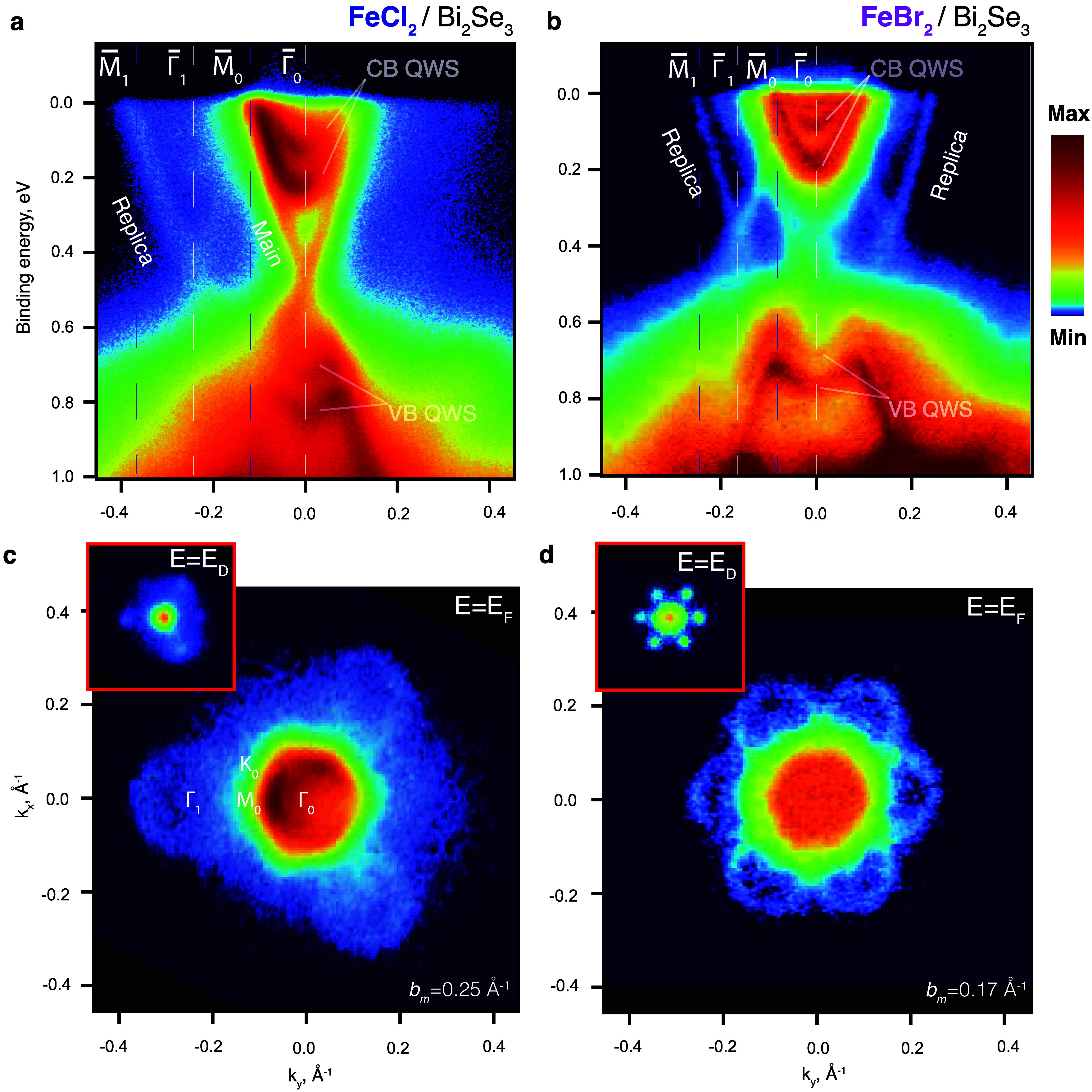
Band structure of FeX_2_ on BS. (a) ARPES dispersion relations of 1 ML (nominal) FC on BS, grown at the STM chamber and transferred to the ARPES end-station in a UHV suitcase. (b) Similar data set for 1 ML (nominal) FB on BS, but this time grown *in situ* at the ARPES system. ARPES constant energy maps for 1 ML FC (c) and 1 ML FB (d) on BS were taken at the Fermi level within the energy window of 50 meV. The color scale is logarithmic. Photon energies are 17 (a, c) and 27 (b, d) eV. Measurements were performed at 17 K along the M̅−Γ̅–M̅ direction in the Brillouin zone (BZ).

In the FC system (Figure 2a), we determine the Dirac point position (*E*_D_), to be around 0.5 eV for the replicas. However, the *E*_D_ for the main cone and the replicas does not coincide, as the uncovered parts of the surface contribute differently than the TMDH-covered regions to the ARPES signal. The Dirac point energies strongly depend on the film preparation conditions and surface aging for both pristine and replicated bands (details in the Supporting Information (Figure S5)). For the FB system (Figure 2b), the Dirac point of the replicas is significantly closer to the Fermi level than that in the main cone. We attribute this difference to the surface fraction of uncovered BS, which is more susceptible to “aging” than the FB-covered one. This is then reflected in a larger downshift of *E*_D_ of the main cone. Another difference is that in the FC system, the replica on the left is more pronounced than the one on the right (Figure 2a), whereas the intensity is fairly symmetric in the FB system (Figure 2b). This is a result of the trigonal symmetry of the formed moiré pattern, also visible in STM (Figure 1e), combined with the matrix elements involved in the photoemission process. The symmetry of the moiré band structure is visualized better in the ARPES constant energy maps shown in Figure 2c,d. For the FC heterostructure (Figure 2c), one can see the bright Fermi surface of the main Dirac cone at the center with QWS states inside and three replicated Fermi surfaces, shifted by the wave vector *b*_*M*_ = 0.25 Å^–1^ and rotated by 120° relative to each other. Closer to the Dirac point, the other three replicas also become visible (see the inset of Figure 2c). For the FB system (Figure 2d), we clearly see all 6 replicas both at the Fermi surface and Dirac point energy with similar intensities, demonstrating an overall hexagonal symmetry, modulated by the photoemission matrix elements.

In the moiré systems formed by twisted graphene or other topologically trivial layers, the replicated bands always avoid crossings, forming the minigaps and eventually resulting in a “flat band”.^37,38^ However, topological surface states are spin-momentum locked^42^ and their crossing should be protected at time-reversal symmetry points. Theoretically then, if the time-reversal symmetry is not broken (nonmagnetic moiré potential), the crossings should be protected at M̅_*i*_ points, but gapped at all other wave-vectors, forming the highly anisotropic minicones centered at the M̅_*i*_ points. However, if the moiré potential is magnetic, as in the case of a TI interfaced with a magnetic insulator, in ref 43, it is predicted that the M̅_*i*_ points are no longer protected and should become gapped. Depending on the specific magnitudes of the scalar and exchange parts of moiré potential, the gaps that existed at other crossings in the purely nonmagnetic case might close with the addition of magnetism. For example, if the moiré exchange term is equal to the scalar potential, the gapless moiré Dirac cones would reemerge at K̅_*i*_, while the M̅_*i*_ points would become gapped. Note that this effect does not depend on the uniform exchange term, which can be zero, keeping the main Dirac point at Γ̅ gapless.

In Figure 3, we present the high-resolution ARPES Fermi surface (a) for 1.5 ML of FB on BS and cuts at several points of the BZ passing through the main Γ̅ (b), K̅_*i*_ (c), and M̅_*i*_ (d) points, taken at the temperature of 2 K, i.e., well below the magnetic transition temperature of FB. These data were obtained on the sample with a significantly reduced area of uncovered parts, which allows one to study the crossing points in detail. We can see that the main Dirac point at the Γ̅ is gapless (b) and thus conclude that the uniform exchange potential is negligible. Crossing of the replicas at the K̅_*i*_ points (c) takes place very close to the Fermi level, making it difficult to resolve the gap opening within our resolution (5 meV). At the M̅_*i*_ points (d), we can see the broadening of the peak by 15–20 meV relative to the equivalent one, but taken far away from the crossing. This is a strong evidence for the minigap opening in the case when the line width of the state is larger than the gap (e).

**Figure 3:**
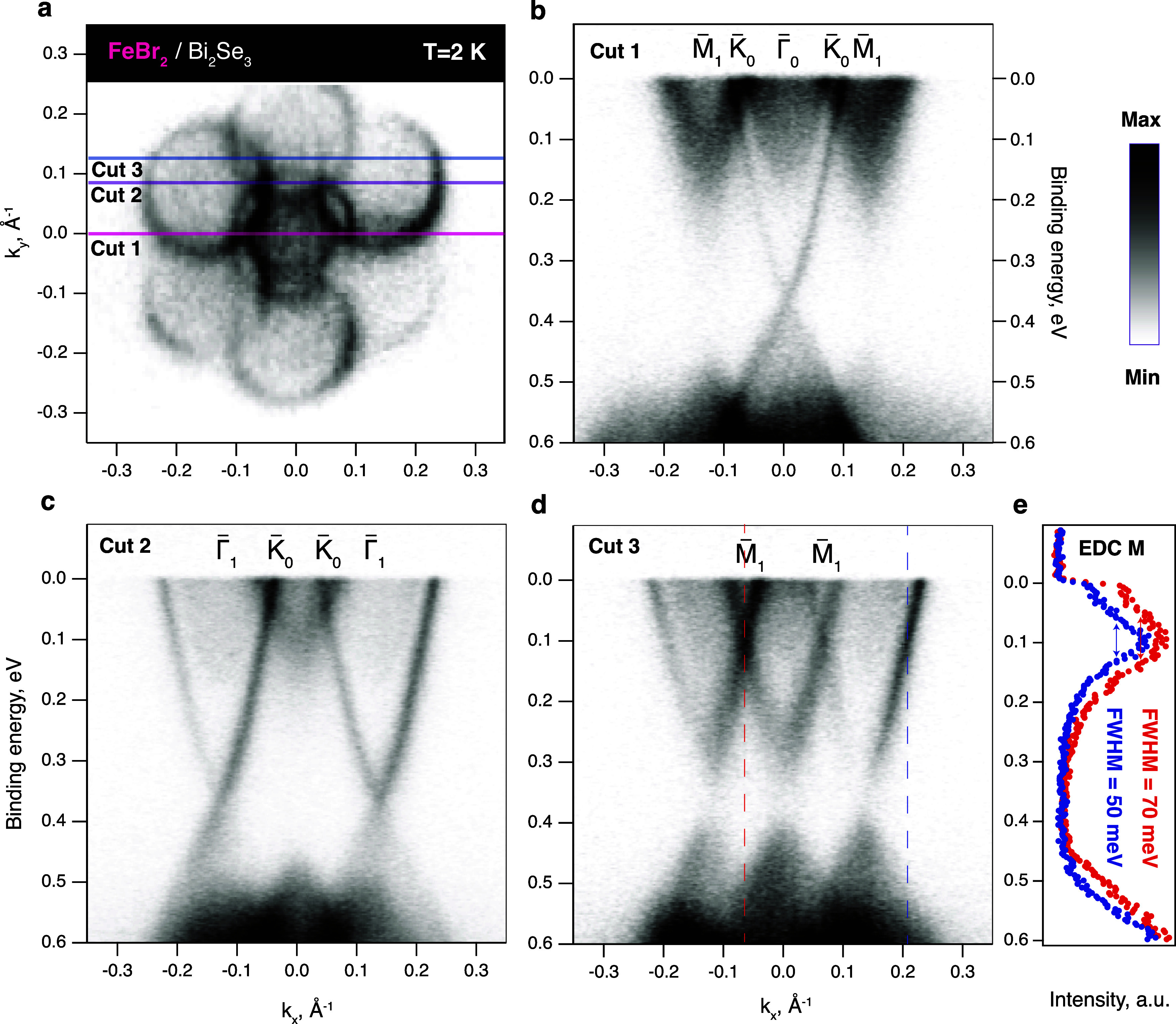
Electronic structure of 1.5 ML of FB on BS. (a) ARPES constant energy map taken at the Fermi level within the energy window of 40 meV. (b–d) ARPES cuts through the main Γ̅ (b, Cut 1), Γ̅–K̅–K̅−Γ̅ points (c, Cut 2) and (b) through M̅ points (d, Cut 3). (e) EDCs taken at the crossing M̅ point (red) and far from the crossing (blue). Spectra were acquired at 2 K by using a photon energy of 27 eV and circular negative polarization.

Such behavior is expected for the magnetic moiré potential acting on the topological surface states. In accordance with the theory,^43^ we speculate that in our system, the exchange and scalar moiré potentials are of similar values of the order of 10 meV. Absence of the gaps at the Γ_*i*_ points indicates that the uniform exchange term is close to zero.

The scenario described above implies the specific moiré exchange interaction induced by the TMDH film on the surface of BS. At this point, we should note that the magnetic structure of the single monolayers of TMHDs is still under debate. Bulk TMDHs are antiferromagnets with relatively low Neel temperatures (25 K for FC and 14 K for FB) and ferromagnetic ordering in 2D layers.^44^ The theoretical prediction for the single monolayer limit is that the Curie temperature could rise up to 210 K for FB and 165 K for FC.^45,46^

Experimentally, the transition temperature of 25 K has been reported for FC monolayer on Au(111), but without the hysteresis loop.^36^ On the other hand, the MOKE experiments on the same heterostructure indicate that the transition temperature might be up to 140 K.^47^ Furthermore, one can expect the magnetic fluctuations to be increased when approaching the 2D limit, which could keep the moiré exchange potential turned on even above the critical temperatures. Thus, except for the agreement that single TMDH monolayers are magnetic, the details of that ordering are still under debate.

We note that the intensity of replicas and amplitudes of minigaps in ARPES depend on the corrugation, potential strength, and photoelectron diffraction.^48,49^ Similar situations have been at play in cuprate high-temperature superconductor Bi_2_Sr_2_CaCu_2_O_8+δ_. In that material, the mismatch between the constituent Bi–O and Cu–O planes results in a one-dimensional (1D) structural supermodulation, essentially a 1D moiré lattice, producing the “supermodulation replicas” visible in the electronic structure.^50^ However, these replicas were interpreted as a diffraction effect of the outgoing photoelectrons originating from Cu–O planes on the supermodulated crystal. It was thought that the supermodulation potential did not affect the electronic states inside the Cu–O planes. Only recently, after improvements in resolution, has it become possible to detect the hybridization gaps and uncover the direct effect of the modulation potential on the states inside the Cu–O planes.^51^ After that, the exciting effects on the superconducting gap have been observed, essentially allowing the sign of the superconducting order parameter to be detected in ARPES.^52^ Although we cannot exclude the diffraction scenario in FeBr_2_/Bi_2_Se_3_, we believe that TSS are affected by the moire potential of the order of 10 meV that may enhance correlations and, according to the theory, lead to superconductivity. We hope that future studies on better tuned film/substrate systems (larger moiré, lower Fermi velocities) accompanied by better experimental resolution will shed light on these intriguing aspects.

## Conclusions

In summary, we have grown and studied 2D magnetic insulators on a topological insulator displaying a homogeneous moiré superlattice. The moiré periodicity and symmetry can be easily tuned by means of changing the film composition. Photoemission data show the replicated Dirac bands crossing at the K̅_*i*_ points of the mini Brillouin zones, while the signatures of the Umklapp gap at M̅_*i*_ points were found. This behavior is in agreement with a magnetic moiré potential acting on the topological surface states, which breaks the time-reversal symmetry resulting in correlated topological phases.

Our study marks the beginning of the experimental topological moiré physics and demonstrates its possibilities. Future investigations of related topological moiré heterostructures are necessary to generate heavy topological Dirac cones with saddle points and enhanced density of states. This, in turn, could enhance correlations and provoke instabilities such as topological superconductivity or interaction-driven insulating states.^15,16^ While in the pure BS the surface electron–phonon coupling has been shown to be anomalously weak,^53^ the enhanced DOS in its moiré counterpart could mediate surface superconductivity. Further, magnetic proximity allows us to tune the minigaps and results in the emergence of topological Chern bands which host higher-order van Hove singularities.^23^ With variation of magnetic overlayers to generate the moiré superlattices on TIs, a completely new field of magnetic TI moiré physics could arise, with its own exciting possibilities.

## Experimental Methods

Iron dichloride (FeCl_2_) and dibromide (FeBr_2_) monolayers were grown on BS using anhydrous beads from Sigma-Aldrich with a purity of 99.99% and a Knudsen cell evaporator with quartz crucibles. The BS surface was prepared *in situ* by exfoliation in ultrahigh vacuum (UHV). During the deposition of FeX_2_ at 10^–9^ mbar, the substrate was constantly heated to ≈100 °C to ensure a large area island growth, due to the increased mobility of FeX_2_ on BS. The amount of the evaporated material was estimated before the growth by using a quartz crystal microbalance (QCM), while low-temperature scanning tunneling microscopy (LT-STM) and low-energy electron diffraction (LEED) were used for the final calibration. The STM and STS experiments were performed using a commercial Scienta-Omicron LT-STM instrument at 4.9 K and 10^–11^ mbar base pressure. The STS spectra and d*I*/d*V* maps were obtained through a lock-in amplifier with an oscillation frequency of *f*_osc_ = 817.3 Hz and a modulation amplitude of *V*_RMS_ = 10.0 mV. ARPES measurements were performed at the LOREA beamline at ALBA with a base pressure of 10^–10^ mbar and the 1-cubed beamline at BESSY with a base pressure of 10^–11^ mbar. The ARPES measurements were done on samples precharacterized in the LT-STM setup and in some cases transferred using a UHV suitcase (p_base_ = 10^–9^ mbar) (FC sample in Figure 2(a)). Separate samples were grown *in situ* and characterized by ARPES at the beamline (FC samples in Figure S6, FB samples in Figures 2(b) and 3), showing good reproducibility. The total energy resolution (at *hν* = 27 eV) was ∼15 meV at LOREA (5 meV at 1-cubed). The angular resolution was ∼0.1 and ∼0.3° along the slit and perpendicular to it, respectively.

## Supplementary Material



## Notes

LEED and additional STM and ARPES data with corresponding description (PDF)
