# Supplementary material for: Emergence of Moiré Dirac Fermions at the Interface of Topological and 2D Magnetic Insulators
Source: ACS Nano. 2025 Oct 10;19(41):36411–8. doi: 10.1021/acsnano.5c10193 (PMC12548352; doi:10.1021/acsnano.5c10193)
Supplement: Supplementary file 1 [file nn5c10193_si_001.pdf]

Supplementary Information for:

Emergence of Moiré Dirac Fermions at the Interface of Topological and 2D Magnetic Insulators

Ilya I. Klimovskikh,[*,†] Sebastien E. Hadjadj,[‡] Amitayush Thakur,[†,¶,§] Aymeric Saunot,[†,¶] Celia Rogero,[†,‡] Massimo Tallarida,[‖] Ji Dai,[‖] Vesna Mikšić Trontl,[⊥] Andrew P. Weber,[†,#] Genda D. Gu,[@] Jorge Lobo-Checa,[*,△,∇] Maxim Ilyn,[*,‡] and Tonica Valla[*,†,⊥]

†*Donostia International Physics Center (DIPC), 20018 Donostia-San Sebastián, Spain*
‡*Centro de Física de Materiales CSIC-UPV/EHU, Donostia-San Sebastián, 20018 Spain*
¶*Departamento de Polímeros y Materiales Avanzados, Universidad del País Vasco UPV/EHU, 20018 Donostia-San Sebastián, Spain*
§*Université Paris-Saclay, CNRS, Institut des Sciences Moléculaires d'Orsay, 91405, Orsay, France*
‖*ALBA Synchrotron Light Source, Cerdanyola del Vallès, 08290 Barcelona, Spain*
⊥*Institut za Fiziku, Bijenička 46, HR-10000 Zagreb, Croatia*
#*ICFO-Institut de Ciencies Fotoniques, The Barcelona Institute of Science and Technology, 08860, Castelldefels (Barcelona), Spain*
@*Condensed Matter Physics and Materials Science Department, Brookhaven National Laboratory, Upton, New York 11973, USA*
△*Instituto de Nanociencia y Materiales de Aragón (INMA), CSIC-Universidad de Zaragoza, 50009, Zaragoza, Spain*
∇*Departamento de Física de la Materia Condensada, Universidad de Zaragoza, E-50009 Zaragoza, Spain*

E-mail: ilya.klimovskikh@dipc.org; jorge.lobo@csic.es; maxim.ilin@ehu.es; tonica.valla@dipc.org

This document contains:

- Description of LEED images.
- Additional details of the STM/STS experimental datasets.
- Details of Resonant ARPES spectra.
- Additional information on the ARPES data.
- Supplementary references.
- 5 supplementary figures referenced in the main text.

LEED images

In Fig. S1 the LEED images of 1 ML $FeCl_2$ and $FeBr_2$ on Bi_2Se_3 are shown. The measurements were performed at the LOREA beamline at ALBA synchrotron. The moiré patterns with different periodicities are evident.

Additional STM/STS datasets

In Figs. S2a and b the STM images of $FeCl_2$ on Bi_2Se_3 for different bias voltages are displayed. In panel a triangular pattern can be observed by measuring with a bias voltage of -0.25 V, which disappears in b where a bias voltage of -0.05 V was applied. In panels c and d the corresponding dI/dV maps at the same biases as in a and b, are shown, respectively. Here no difference between the two patterns is visible and the orientation of the moiré pattern matches the one shown in b. This indicates that the $FeCl_2$ film on Bi_2Se_3 is very uniform. In panel e the STS spectra for the clean Bi_2Se_3 and $FeCl_2$ are displayed. By comparing the two spectra no major difference besides the appearance of a broad peak for the $FeCl_2$ at around -0.23 V.

In Fig. S3a the topographic image of approximately 0.5 ML $FeCl_2$ on Bi_2Se_3 is shown, with large islands of $FeCl_2$ and uncovered regions of Bi_2Se_3. In panels b and c the topographic and atomic-resolution images of the uncovered Bi_2Se_3 substrate are displayed. The extracted lattice constant of Bi_2Se_3 matches the theoretical value of 4.14 Å.[1] In panel d the STS spectra of the uncovered regions for $FeCl_2$ and $FeBr_2$ on Bi_2Se_3 systems are presented. As evidenced in Fig. S3d, the minima marking the Dirac points of the pristine regions are found at different energy positions. In particular, the substrate used to grow the $FeCl_2$ shows the minimum at -0.23 V, whereas for the substrate used for the $FeBr_2$ is located at -0.38 V. Such difference is associated with different Bi_2Se_3 crystal batches and therefore requires a shift of $+0.15$ V applied to the $FeBr_2$ STS to align their Dirac points and be directly compared. This energy shift is applied in Fig. 1f of the main text. It is important to stress that such different batches

were not used in any of the ARPES experiments.

Resonant ARPES spectra

In order to have a better insight into the electronic structure of the $FeCl_2$ on Bi_2Se_3 and to identify the character of the observed states we have performed the photoemission measurements under Fe $3p-3d$ resonant conditions (Fig. S4b) and off resonant for comparison (Fig. S4a). In Fig. S4a we can see the strongly dispersing valence band states of Bi_2Se_3, that are overlapping with two less dispersive states at binding energies higher than 2 eV. Under the Fe resonant conditions the non-dispersive states become much more intense, that points to their $FeCl_2$ origin. The resonance intensity enhancement is clearly seen in panel (c), where EDCs around Γ point are presented. The ARPES spectra are therefore in a good agreement with the STS data in Fig. 1 of the main text.

Additional notes on the ARPES data

ARPES data for three FC samples with different coverages and growth conditions are shown in Fig. S5. In panel a one can see the Dirac point position is around 0.5 eV, and it is the same for the replicated one. Reducing the coverage of $FeCl_2$ down to 0.5 ML (see panel b), leads to decrease of the replica intensity, relative to the main Dirac state. The Dirac point position of replica is around 0.45 eV on this image, while for the main one it is higher. This difference is most probably related to additional signal to the main band, coming from film uncovered parts. The panel c shows spectra for 1 ML of the film, but studied right after the synthesis, and we can see the Dirac point positions of 0.35 eV for both main and replicated Dirac bands, and also the second Dirac cone below the main one, shifted by 0.1 eV towards higher BE, which obviously comes from the uncovered parts of the sample illuminated by the incident beam.

References

1. Mazumder, K.; Shirage, P. M. A brief review of Bi_2Se_3 based topological insulator: From fundamentals to applications. *Journal of Alloys and Compounds* **2021**, *888*, 161492.

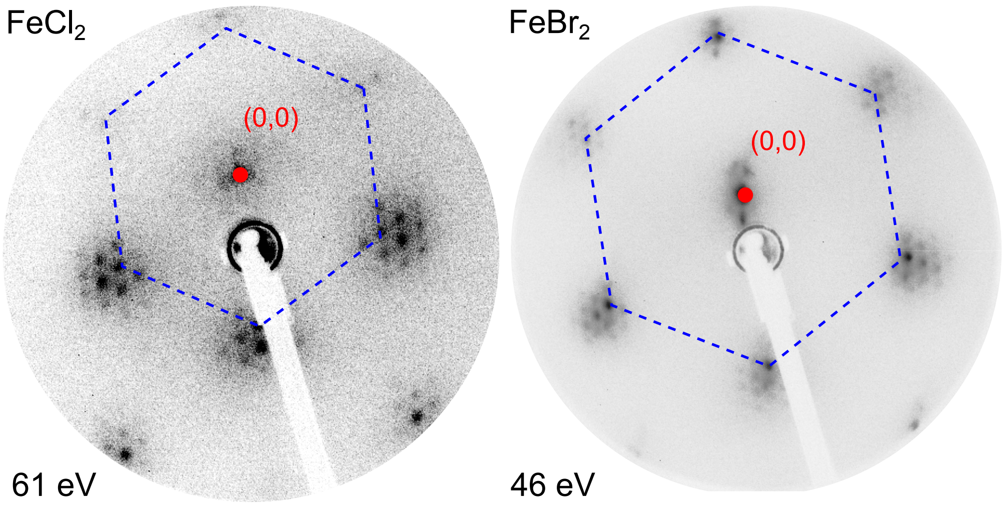

Figure S1: LEED images of 1 ML $FeCl_2$ (left) and $FeBr_2$ (right) on Bi_2Se_3. In both images the hexagonal LEED pattern of the substrate is indicated by the blue hexagon. In the center the (0,0)-spot is indicated (red dot).

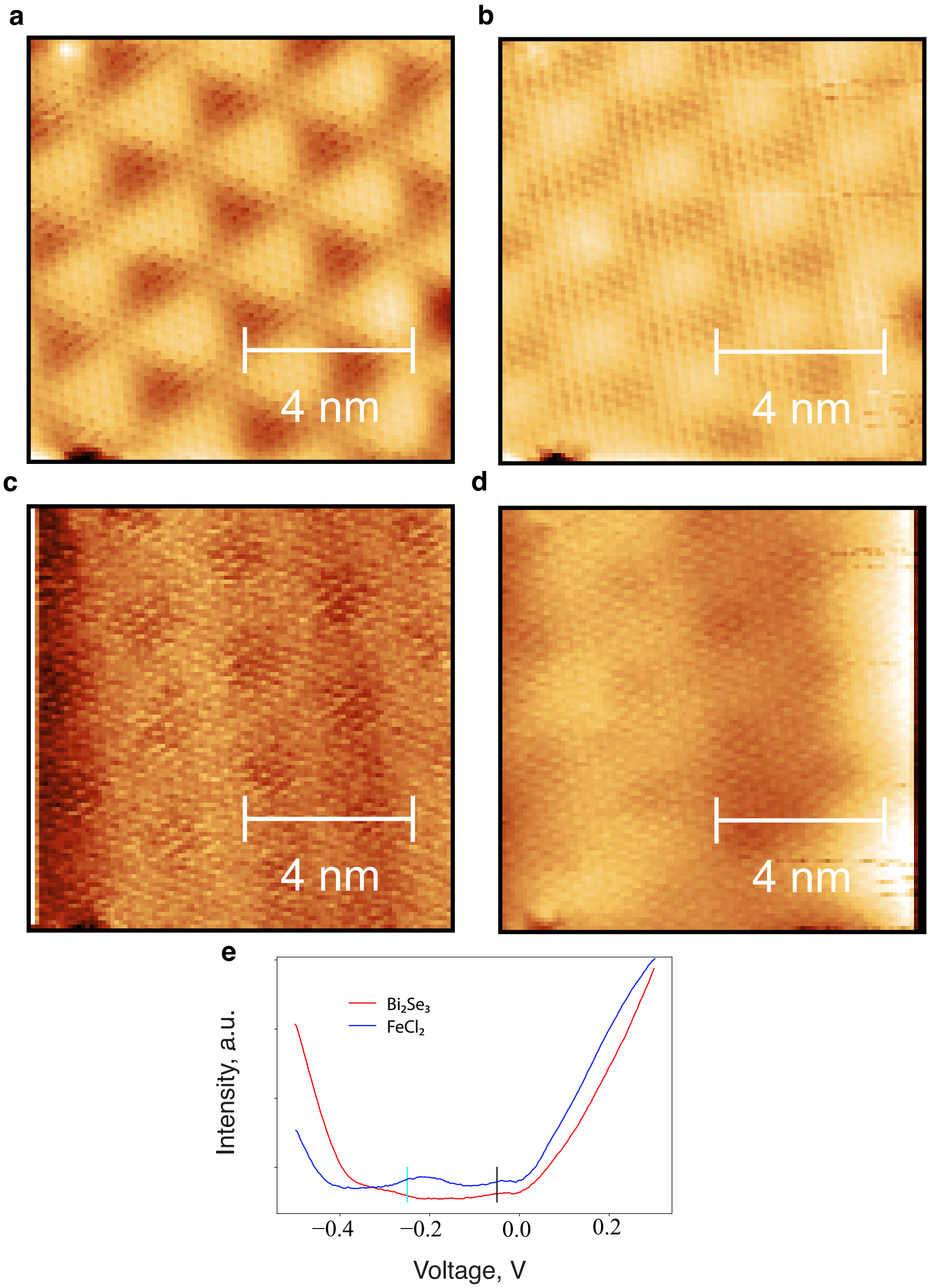

Figure S2: STM images of $FeCl_2$ on Bi_2Se_3 measured at $U_{bias} = -0.25$ V (a) and $U_{bias} = -0.05$ V (b) with a $I_T = 50$ pA. In (c) and (d) the corresponding dI/dV images for (a) and (b) are shown. Here no difference of the observed pattern is visible. In (e) the STS spectra of clean Bi_2Se_3 and $FeCl_2$ are shown. The vertical lines are indicating the two used bias voltages in (a) and (b).

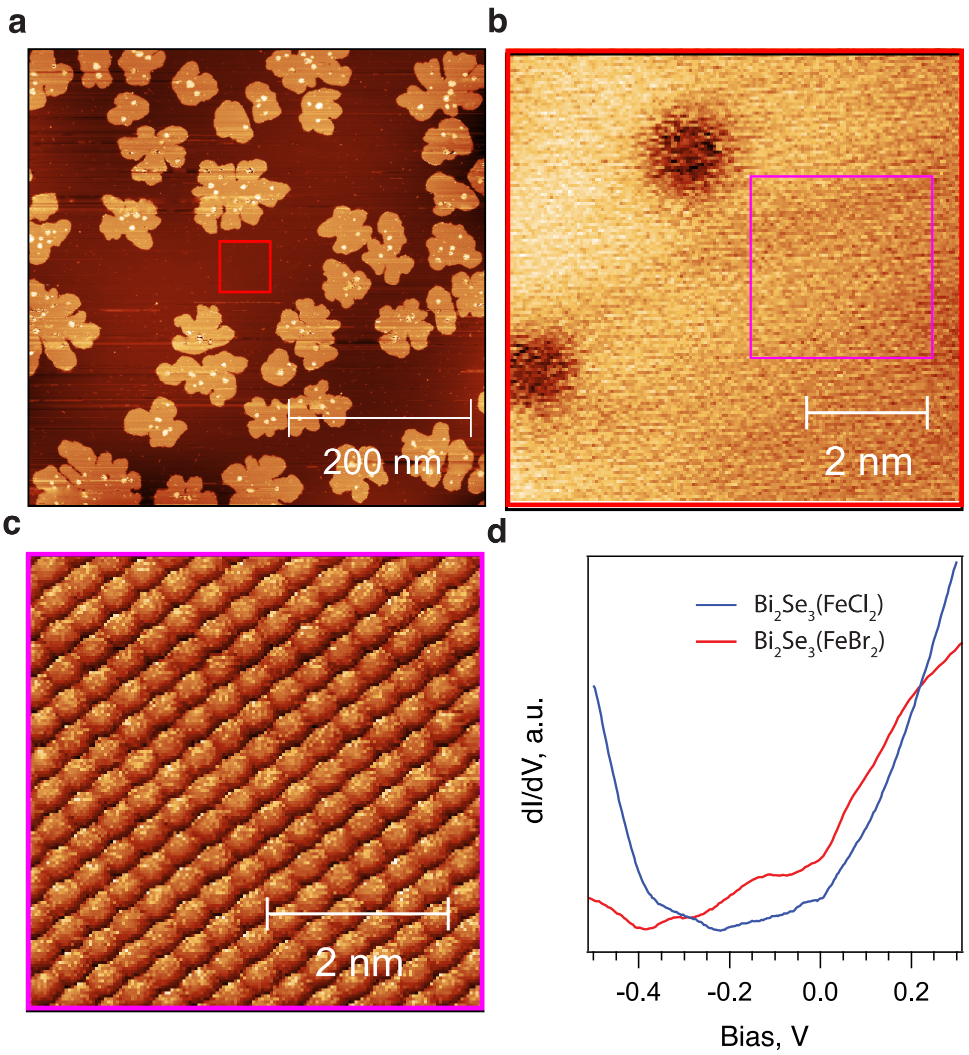

Figure S3: (a) Topographic STM image of approximately 0.5 ML of $FeCl_2$ on Bi_2Se_3. (b) topographic image of the uncovered Bi_2Se_3 surface from the red square in (a). (c) Atomic resolution of Bi_2Se_3 from the region marked by the pink square in (b). (d) Average STS measurement of Bi_2Se_3 surface at the two different substrates used for the film growth. The minima is visibly shifted by 0.15 V due to the different n-doping existing between the two substrates. (a) $U_{bias} = 1.6$ V and $I_T = 5$ pA, (b) $U_{bias} = -1.0$ V and $I_T = 5$ pA, and (c) $U_{bias} = 0.07$ V and $I_T = 160$ pA.

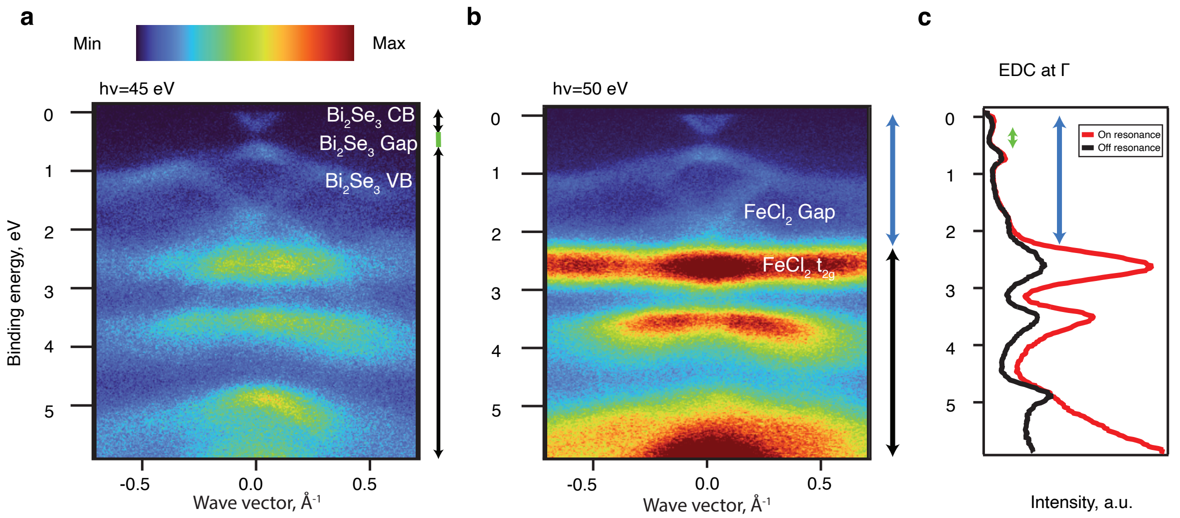

Figure S4: ARPES spectra of 1 ML of $FeCl_2$ on Bi_2Se_3 taken at a photon energy of 45 eV (off resonance, a) and 50 eV (on resonance, b). (c) Energy distribution curves taken at the $\bar{\Gamma}$ point for both photon energies. The measurements has been performed at 17 K.

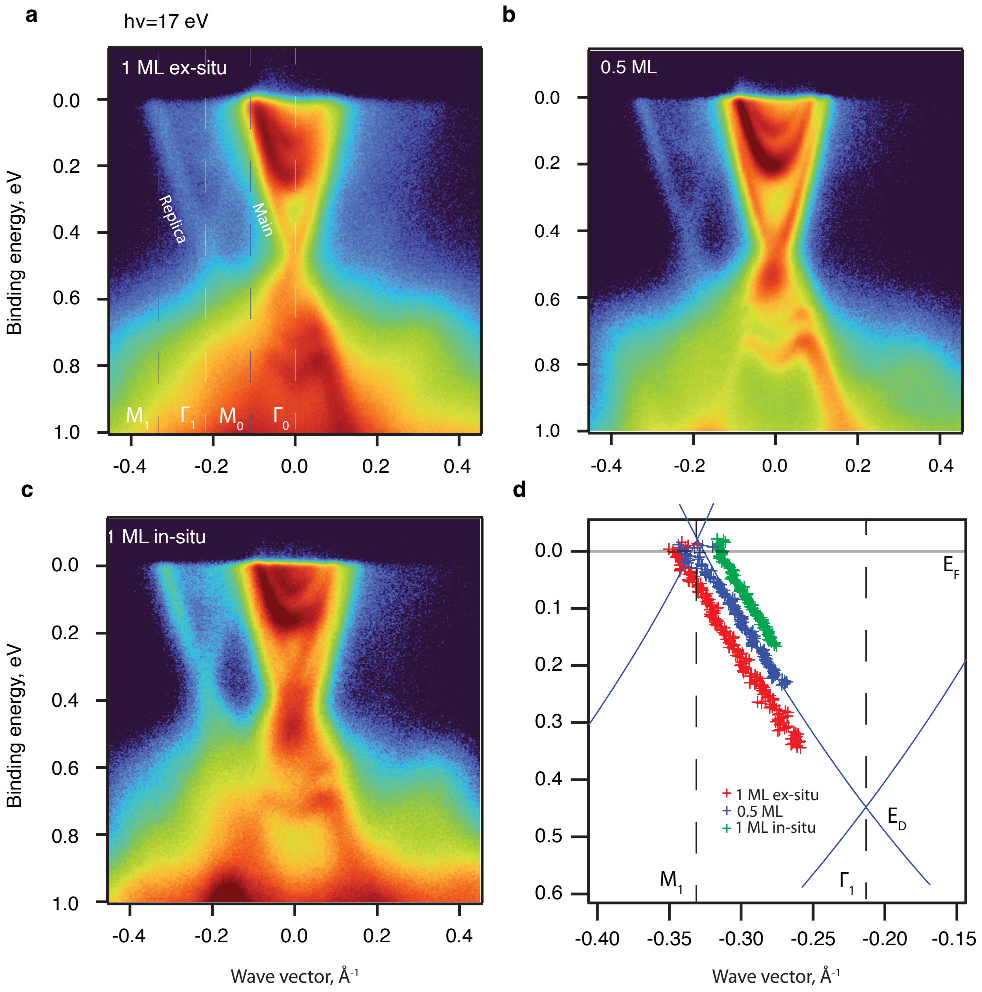

Figure S5: (a) ARPES dispersion relations of 1 ML $FeCl_2$ on Bi_2Se_3, that was transfered in UHV case to the synchrotron ARPES end-station. b) The same for the 0.5 ML coverage grown in-situ. c) The same for 1 ML coverage, but grown in-situ. d) Extracted positions of the replicated band peak for these three systems, obtained as lorentian fitting of the momentum distribution curves (MDCs). Measurements were done at 17 K along the $\bar{\mathrm{M}} - \bar{\Gamma} - \bar{\mathrm{M}}$ direction in the BZ.
